# Current dietary intake of the Japanese population in reference to the planetary health diet-preliminary assessment

**DOI:** 10.3389/fnut.2023.1116105

**Published:** 2023-04-03

**Authors:** Marika Nomura, Miwa Yamaguchi, Yuji Inada, Nobuo Nishi

**Affiliations:** ^1^School of Tropical Medicine and Global Health, Nagasaki University, Nagasaki, Japan; ^2^International Center for Nutrition and Information, National Institutes of Biomedical Innovation, Health and Nutrition, Osaka, Japan; ^3^Human Development Department, Japan International Cooperation Agency, Tokyo, Japan; ^4^The African Union Development Agency, New Partnership for Africa’s Development, Midland, South Africa

**Keywords:** sustainable healthy diets, planetary health diet, Japan National Health and Nutrition Survey, diet gap, protein food intake, red meat

## Abstract

**Introduction:**

We sought to assess the Japanese diet by examining the current dietary intake in Japan using the global reference diet from the EAT-Lancet Commission (Planetary Health Diet; PHD), from the perspective of protein intake in different age groups.

**Methods:**

Average dietary intake by food group in the Japan National Health and Nutrition Survey 2019 (NHNS 2019) was converted to the PHD food groups, and the diet gap (DG) (%) of the global reference of the PHD was calculated by age group.

**Results:**

Although the DG of the intake was excessive compared with the global reference of the PHD in most food groups in all age groups (7.1–416%), the intake exceeded the upper limit of the range only for red meat (640%). Red meat had the highest DG among subjects in their 40s, although the DG decreased with increasing age. Protein intake was within the possible range and did not greatly exceed the recommended dietary intake in the Japanese standard.

**Discussion:**

The current Japanese diet contains an excessive intake of red meat in terms of the global reference of the PHD. This trend is similar to that previously reported in various western regions and countries. However, the Japanese diet does not significantly exceed the recommended protein intake for Japanese people, suggesting that the PHD is an environmentally friendly and healthy choice for younger and older age groups in an aging Japanese society. Policy makers need to develop sustainable and healthy food-based dietary guidelines in addition to providing food and nutrition education and developing a food environment that encourages sustainable and healthy choices to support dietary change.

## Introduction

Obesity is on the rise in most countries and regions worldwide; approximately 1.9 billion adults are overweight or obese, while 462 million are underweight (1). The “double burden of malnutrition” during this nutrition transition process has become an urgent health issue in all countries and regions (2). One approach for addressing this increasingly complex nutritional problem is the promotion of a healthy diet (3). A healthy diet can help prevent all forms of malnutrition and diet-related non-communicable diseases (NCDs), such as diabetes, heart disease, stroke, and cancer (3). In addition to the double burden of malnutrition, a triple burden of malnutrition has been proposed. The three pandemics of undernutrition, obesity and climate change, represent a “global syndemic” with the potential to affect most people in all countries and regions of the world (4). If the health of the global food production system, which is susceptible to the negative impacts of climate change, is not maintained, the impact of undernutrition, overnutrition, and diet-related NCDs will be increased by a large magnitude (5).

This series of concepts has led to the development of the Sustainable Healthy Diets (SHDs) guidelines in “Sustainable Healthy Diets-Guiding Principles” published by the Food and Agriculture Organization (FAO) and the World Health Organization (WHO) (6). SHDs are “dietary patterns that promote all dimensions of individuals’ health and wellbeing; have low environmental pressure and impact; are accessible, affordable, safe and equitable; and are culturally acceptable.” The Planetary Health Diet (PHD) is a specific diet that embodies concept of the SHD, and was released by the EAT-Lancet Commission on Healthy Diets from Sustainable Food Systems (EAT-Lancet) (7). The PHD is a global reference diet for adults that is optimal for human health and environmental sustainability. The PHD emphasizes a plant-based diet that includes whole grains, fruits, vegetables, nuts, and legumes, but also eating minimally processed foods and nutrient-rich plant foods daily. Meat and dairy products are an important part of the diet, but in much smaller proportions than plant foods. Dietary goals require 2,500 kcal/day for the average adult. This amount varies with age, gender, activity level, and health status, but overconsumption is a costly waste of food in terms of both human health and the environment (7). This report estimated that by adhering to the PHD, approximately 11 million deaths per year could be averted and premature deaths could be reduced by 19% by 2030. The report also reported the diet gap (DG) of the intake for each food group by region. In East Asia and the Pacific, red meat, starchy vegetables, eggs, and fish were indicated to exceed 100% of DG. However, no results were available for Japan alone, and the PHD does not completely match the Japanese dietary intake.

The principles of the PHD not only relate to what is nutritionally consumed, but also to how the food is produced and distributed globally. Thus, nutrition improvement is important for each goal, especially SDGs Nos. 2, 3, 6, 11, 12, 13, 14, and 15. Additionally, it is globally recognized that Goal 17 (“Partnership”) is particularly important because improving people’s nutrition requires the participation of all sectors and stakeholders involved in food and diet. Thus, in response to the Sustainable Development Goals, a healthy diet should be considered for potential benefits to the planet, because human health and environmental sustainability are important topics for the 17 goals (8).

Since the publication of the SHDs and the PHD in 2019, diets in various countries have been examined for their feasibility (9). For example, in Italy (10, 11), the United States (12), India (13), Mexico (14), Spain (15), and Australia (16), the EAT-Lancet Reference Diet was evaluated for its feasibility of achievement. Although the foods for which intake was found to be inadequate or excessive differed according to each country’s dietary pattern, the intake of animal protein was found to be excessive in every country with the exception of India. The health risks posed by a high intake of red meat and processed meat consumption are associated with type 2 diabetes, coronary heart disease, stroke, and certain cancers, as well as total mortality (17). Because red meat is classified as a high environmental impact food (18), it is important to consider how much and how it is consumed, particularly in the future. A characteristic feature of the PHD is a substantial reduction in the intake of animal-based protein such as red meat; as shown in the PHD summary report, the current global DG of red meat is 288% of the global reference (7).

The Japanese diet, along with the Mediterranean diet, is highlighted as an example of a sustainable and healthy diet (6, 19). Historically, Japan overcame postwar undernutrition, and adopted various nutrition policies. In the past, Japan also used to have a diet that consisted mainly of carbohydrates. In the modern era, dietary patterns have changed, and fat and protein intake has increased. Japan currently has one of the world’s longest life expectancies and has controlled the increase in obesity; however, the consumption of red meat has increased in recent years (20), and diet-related NCDs are the leading cause of mortality. As an island nation that is vulnerable to climate change, and with a food self-sufficiency rate of less than 40%, it is necessary to consider how to proceed with SHDs. However, there is a lack of basic research examining this issue, and it remains unclear whether the current protein intake is sufficient or insufficient for the aging Japanese population, from an environmental perspective. In Japan, based on the Health Promotion Act (Act No. 103 of 2002), the National Health and Nutrition Survey (NHNS) is a survey conducted annually to understand the status of health, nutritional intake, and lifestyle habits in the population, and to obtain basic data necessary for comprehensive health promotion. The tabulated results are publicly available and can be used by any user. Thus, the purpose of the current study was to compare the global reference of PHD with the common diet patterns from the tabulated results of the NHNS, from the perspective of protein intake in different age groups.

## Materials and methods

### The planetary health diet

The global reference of the PHD specifies half of the daily dietary intake in the form of fruits and vegetables, and the other half in the form of whole grains, tubers and starchy vegetables, dairy foods, protein sources, added fats and added sugars (Table 1) (7). The referenced intake is presented as a macronutrient intake (g/day) and possible range (g/day). To calculate these amounts, the PHD employs a total daily energy intake of 2,500 kcal/day, with modifications for sex, body mass index, and physical activity.

**Table 1:** Food group and dietary intake of National Health and Nutrition Survey, the global reference of the Planetary Health Diet and diet gap.

Planetary health diet (PHD)		Japan National Health and Nutrition Survey^b^			DG (%)^e^
Food group	Global reference (g/day)^a^	Food groups	Food items	Intake (g/day)^c^	
Whole grains	N.A.	–	–	N.A.	N.A.
Tubers or starchy vegetables	38 (0, 77)	Potatoes	Potatoes, potato products, processed starch	50 (67)	131
Vegetables	230 (153, 459)	Vegetables, mushrooms, and seaweeds	Green and yellow and other vegetables, vegetable juice, salted or pickled in vegetables, mushrooms, and seaweeds	309^d^ (180)	134
Fruits	153 (77, 230)	Fruits	Fresh fruit, jam, fruit juice	100 (129)	65
Dairy foods	191 (0, 383)	Diary (milk and dairy products, other milk products)	Milk, cheese, and yogurt, other dairy products, other milks	111 (141)	58
Beef, lamb, and pork	11 (0, 21)	Meat (livestock)	Beef, pork, ham and sausages, other meats	69 (68)	640
Chicken and other poultry	22 (0, 44)	Meat (poultry)	chicken, other poultry	31 (56)	139
Eggs	10 (0, 19)	Eggs	Eggs	41 (39)	416
Fish	21 (0, 77)	Fish and shellfish	Raw fish and shellfish, fish, and shellfish products	69 (72)	319
Legumes	57 (0, 77)	Beans	Soybeans and soy products and other products of legumes	65 (81)	112
Nuts	38 (0, 57)	Seeds and nuts	–	2.7 (10)	7.1
Unsaturated oils	31 (15, 61)	Oils and fats	Margarine, vegetable oils Butter, animal fats	10^d^	33
Saturated oils	9.0 (0, 9.0)			1.4^d^	15
Added sugars	24 (0, 24)	Sugars and sweeteners	–	6.5 (9.0)	27

PHD, planetary health diet; DG, diet gap; N.A., not available. ^a^Average macronutrient intake and possible range of each food group for the 2,500 kcal/day in the PHD was converted by a total energy intake of 1,915 kcal/day in the National Health and Nutrition Survey 2019. ^b^Food groups in the Japan National Health and Nutrition Survey as referenced by the National Health and Nutrition Survey. ^c^Average (standard deviation) dietary intake was referenced by the NHNS 2019. ^d^Average (standard deviation) was 281 (176) in vegetables, 18 (31) in mushrooms, 11 (21) in seaweeds, 1.1 (3.4) in margarine, 8.9 (9.0) in vegetable oils, 1.2 (3.4) in butter, and 0.2 (1.3) in animal fats. ^e^DG (%) = (NHNS dietary intake (g/day)/the PHD macronutrient intake (g/day)) * 100.

### The Japan National Health and Nutrition Survey

The Japan National Health and Nutrition Survey (NHNS) has been conducted over 70 years, and the tabulated aggregate results of the survey are published by the Ministry of Health, Labour and Welfare (21). The dietary intake survey is conducted on a single day in November (excluding Sundays and public holidays) using semi-weighed household dietary records (22). We classified the food groups in the NHNS 2019 (23) on the basis of the PHD food groups (Table 1). This study did not use whole grains because this category was not available in the NHNS. Vegetables, mushrooms, and seaweeds in NHNS were included as vegetables in the PHD. Processed foods were included in the food group with the most contained ingredients. As a supplemental investigation, we conducted the same calculation using the NHNS 2000, 2005, 2010, and 2015 to investigate variation over time. Intake of “beef, lamb and pork,” “chicken and other poultry,” “unsaturated oils,” and “saturated oils” were not available in the NHNS in 2000, 2005, and 2010. Therefore, we indicated the aggregated intake of “meat” and “oils and fats.”

### Statistical analysis

First, we summed the results of each food to calculate the dietary intake of vegetables, unsaturated oil, and saturated oil: vegetables, mushrooms, and seaweeds for the vegetables food group; margarine and vegetable oils for unsaturated oil; and butter, animal fats for saturated oil, in the NHNS 2019. The standard deviation was presented for each item. We calculated the PHD reference diet for Japanese using those results to adjust the global reference of PHD to the current total energy intake of the Japanese population (23). Specifically, the macronutrient intake and possible range (g/day) of each food group relative to the 2,500 kcal/day proposed by the global reference of the PHD were converted to the NHNS 2019 average total energy intake of 1915 kcal. Second, DG (%) was calculated as actual NHNS dietary intake (g/day) divided by the PHD macronutrient intake (g/day). In the same manner, we calculated the PHD macronutrient intake and possible range (g/day) and DG (%) to match the NHNS 2019 average total energy intake by age groups (i.e., 20–29 years, 30–39 years, 40–49 years, 50–59 years, 60–69 years, 70–79 years, and 80 years and older). Possible range (g/day) and DG (%) for the total age groups were calculated on the basis of the NHNS 2000, 2005, 2010, and 2015 as a supplemental analysis. All statistical analyses were conducted using Stata (ver. 15.0; StataCorp, College Station, TX, United States).

## Results

The NHNS 2019 Nutritional Intake Status Survey covered 5,865 individuals. Of these, 4,927 (2,297 men and 2,630 women) were 20 years of age or older and were included in this analysis. By age group, 365 were aged 20–29, 460 were aged 30–39, 742 were aged 40–49, 775 were aged 50–59, 1,046 were aged 60–69, and 1,539 were aged 70 and older.

Table 1 shows the global reference of the PHD, NHNS dietary intake, and DG for each food group. The food groups that exceeded the global reference of PHD were, in descending order, beef, lamb and pork, eggs, fish, chicken and other poultry, vegetables, and tubers or starchy vegetables. The food groups with less than 100% of DG were, in descending order, fruits, dairy foods, unsaturated oils, added sugars, and saturated oils; the lowest DG was for nuts. In terms of DG, eight of the food groups exceeded the reference value, but in terms of possible range, only two food groups (beef, lamb, and pork and fish) indicated excessive intake. Similar trends were observed in the NHNS in 2000, 2005, 2010, and 2015 (Supplementary Table S1).

Table 2 shows the total energy intake, protein intake, and intake of NHNS protein foods by age group. The average total energy intake was 1,898 kcal/day and was highest in subjects in their 60s. Protein intake was highest among subjects in their 70s and lowest among subjects in their 80s. Intakes of red meat such as beef, lamb, and pork were highest in subjects in their 40s and relatively lower in older age groups. Intake of white meat (chicken and other poultry) was highest among subjects in their 20s and lowest among older cohorts. Intake of fish and legumes was higher among older groups, with the exception of subjects in their 80s. The intake of eggs was similar among all age groups.

**Table 2:** Total energy intake, protein intake, and National Health and Nutrition Survey dietary intake of protein foods^a^ by age.

Age group	20s	30s	40s	50s	60s	70s	80s+
Total energy intake (kcal/day)^c^	1900 (664)	1859 (558)	1939 (607)	1918 (581)	1972 (586)	1945 (549)	1750 (517)
Protein intake (g/day)^b^	71 (27)	68 (24)	72 (26)	70 (24)	75 (24)	76 (26)	66 (23)
Food group (g/day)							
Beef, lamb and pork	81 (77)	75 (70)	86 (73)	72 (72)	67 (65)	59 (61)	48 (55)
Chicken and other poultry	49 (68)	40 (63)	43 (68)	34 (57)	26 (49)	21 (45)	18 (39)
Eggs	39 (40)	38 (38)	40 (38)	40 (38)	44 (39)	45 (39)	38 (37)
Fish	51 (58)	51 (62)	53 (65)	59(63)	78 (74)	89 (83)	74 (64)
Legumes	47 (73)	45 (61)	52 (73)	65 (91)	77 (87)	76 (85)	65 (72)
Nuts	1.3 (4.2)	2.9 (17)	2.1 (7.3)	3.0 (12)	3.2 (9.4)	3.2 (9.8)	2.2 (6.2)

^a^Average (standard deviation) of dietary intake was referenced by the National Health and Nutrition Survey 2019. ^b^Protein intake was referenced by the NHNS 2019. ^c^Total energy intake was referenced by the NHNS 2019.

**Figure 1:**
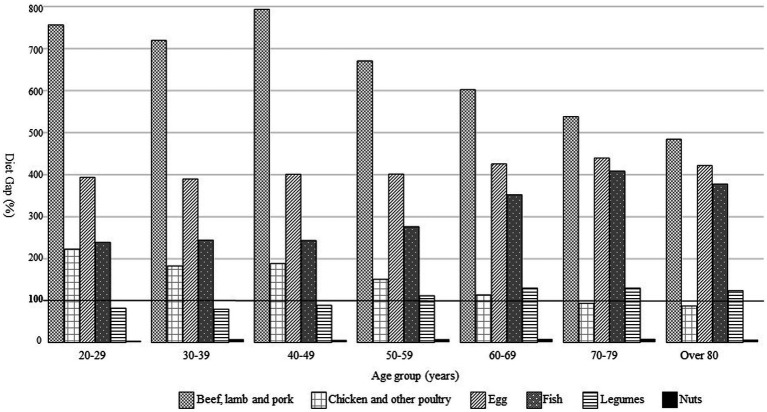
The diet gap between current intakes of protein food in the Planetary Health Diet by age group.

As shown in Figure 1, DG of red meat and eggs far exceeded the upper limit of the possible range of the global reference of PHD in all age groups. Red meat had the highest DG among subjects in their 40s, although the DG decreased with increasing age. The DG for fish increased with age into the 70s. Legume intake did not reach 100% of DG in subjects in their 20, 30, and 40s, but exceeded 100% for those in their 50s and above.

## Discussion

This is the first study to show that in the modern Japanese diet, red meat was far in excess of the upper range limit of the global reference of PHD. According to EAT-Lancet, red meat and starchy vegetables are “limited intake,” eggs, poultry and daily foods are “optional foods,” and fish, vegetable, fruit, legumes, whole grains and nuts are “emphasized foods.” Thus, as shown in this study, similar to the results shown in previous studies from other countries or regions, excessive intake of red meat poses a challenge to achieving the SHDs, even in Japan. From the perspective of the environmental cost per calorie, the effect of beef production on CO2 emissions is much higher than that of other protein sources (24). Therefore, from both health and environmental perspectives, determining how to reduce red meat consumption is a pressing issue (25, 26). Given the impact of meat production on the planet, changing animal protein consumption is a matter of nutrition security. While it is difficult to change consumer dietary behavior, environmental motivations are reported to be useful and proven, especially in Europe and Asia (27). In Asian countries, however, there are still very few similar studies that have assessed country-specific food intake using the PHD reference diet. In China, the average intake of meat, especially pork, continues to increase (28). Taiwan has the third highest percentage of vegetarians in the world, estimated at about 13% of the population (29). In neighboring East Asia, it is expected that dietary patterns will be analyzed using PHD reference diet, as in this study, in order to reconsider the state of meat intake.

Until now, the intake status of meat types has rarely been examined in Japan, from an environmental perspective. Possible explanations for the high intake of red meat in this study include the commonality of pork in the Japanese daily diet (23). In addition, household consumption of raw meat has exceeded that of raw fish in Japan since 2007 (20). Regional differences in meat consumption can be seen, with pork being more popular in eastern regions, while beef and chicken are more popular in western regions (30). However, regional differences have not been observed with regard to high-meat low-fish dietary patterns, compared to high-vegetable dietary patterns and other factors (31). From the global view, the Japanese diet is characterized as having a low red meat intake compared with the Western diet (32). Having said that, the Japanese diet must address red meat intake from an environmental perspective. To reduce the intake of red meat, it would be appropriate to shift from red meat intake to an increased intake of chicken and other poultry, which are relatively lower in fat. In addition, shifting daily intake from animal-based protein foods (i.e., beef, pork, and fish) to plant-based protein foods (legumes) may also be appropriate because of the familiarity of legumes in the Japanese diet (33). Replacing beef with chicken has a positive health impact (34), chicken or even pork should be the preferred choice, because they have significantly reduced carbon and water footprints (35). In addition, shifting daily intake from animal-based protein foods (i.e., beef, pork, and fish) to plant-based protein foods (legumes) is also appropriate not only because of environmental benefit (36), but also the familiarity of legumes in the Japanese diet (33). From an environmental perspective, veganism is the least environmentally impactful diet, and consideration should be given to eating locally produced products and reducing the environmental costs of transportation (37).

The current study suggests the importance of examining PHD feasibility by age group. The results revealed that younger people tended to consume relatively fewer fish and legumes, while more red meat. Older people tended to have a lower intake of chicken and other poultry. For all age groups, consumption of nuts was relatively uncommon in the Japanese diet. Therefore, shifting to an increased intake of fish and legumes for younger people and chicken for older people are plausible options for reducing red meat intake. On the other hand, as shown in Table 2, actual total protein intake was sufficient, and not far in excess of the recommended protein intake (60–65 g for men and 50–55 g for women) for Japanese in all age groups (38). This suggests that although excessive consumption of red meat is a challenge compared with the global reference of PHD, protein intake itself can be sustained at the current level, because it does not significantly exceed the protein intake recommendation for Japanese people in any age group. Historically, as shown in the Supplementary Table S1, in Japan, there has been a change to a less desirable diet with more meat, less fish and less fruits, in these two decades. In fact, when the current older adults were in their middle ages, average fish and seafood intake was higher in Japan than now (20). A study showed comparing the older Japanese people with their children’s age groups, the sufficiency of all nutrients was higher in the older people. It was observed that the Japanese dietary pattern has not been carried over to the next generation (39). Also, a study of the dietary patterns of Japanese people in the year following the Great East Japan earthquake showed that young people and men prefer meat-eating patterns, while the older and women choose prudent traditional eating patterns (40). Taken all together, these implications suggest that the current Japanese older people may be maintaining the dietary pattern of their middle ages. The current young and middle-ages, who have “westernized dietary pattern,” may not necessarily make the same healthy Japanese food choices with appropriate amount as today’s older people, so it is reasonable to target dietary interventions primarily to younger people (41, 42). As noted by Willett, the optimal energy and nutrient intake for maintaining a healthy weight depends on body size and level of physical activity (7). Similarly, because body size and the level of physical activity are also highly dependent on age, it is still necessary to take age into consideration when assessing optimal dietary patterns.

The current findings indicated that a shift from red meat to white meat, and ideally to plant-based protein with consideration of their age, rather than reducing the intake of protein itself, may be a sustainable and healthy option for Japanese. Many essential amino acids can be supplemented with a combination of plant-based protein. Thus, it is reasonable for the Japanese population to sustain their current diet while transitioning to a PHD (43). Importantly, for older population groups, it is necessary to ensure sufficient protein intake to prevent frailty or osteoporosis (44). A study reported that total protein intake, regardless of whether it is animal- or plant-based protein, was significantly inversely associated with frailty in older Japanese women (45). The proportion of obesity (BMI > 30) among Japanese 20 year or above was 5.5% in men and 3.8% in women, NHNS 2019 reported (NHNS 2019 in Japanese version). Since the proportion of obese is not particularly high yet in Japan, this result supports the quantity of protein intake should remain the same while the quality of protein should be changed. These reports support the notion that modifying the type of protein intake is an option, even for the older population, and does not require drastic modifications in dietary pattern. This suggests that even in Japanese society, in which the population is aging, the PHD is an environmentally friendly and healthy choice for both younger and older age groups. However, there are things to consider when making a healthy transition from red meat to white meat. Both red and white meats have higher cholesterol levels than plant protein (46). Similarly, saturated fatty acids are higher in both red and white meat (46). There is no significant impact on hypercholesterolemia for red meat or skinless poultry (47). However, according to food composition tables, chicken breast and chicken leg also differ in fat content (48). Therefore, even when choosing chicken meat, attention should be paid to whether the meat is with or without skin, and which part of the chicken the meat is from.

The results of the current study have implications for policy makers. The Dietary Guidelines for Japan comprise 10 points for maintaining a desirable dietary lifestyle, developed in 2000 in collaboration with several government ministries (49). The main purpose of these guidelines is to address not only the nutritional aspects of maintaining an appropriate weight, but also the enjoyment and self-management of food, environmental issues such as food loss, and aspects of lifestyle and culture. Therefore, the dietary guidelines ultimately resemble the basic concepts presented in the PHD. In 2005, the Dietary Balance Guide “Japanese Food Guide Spinning Top” was published as a guide for the public that translates dietary guidelines into tangible actions (50). The Japanese Food Guide Spinning Top is diet-based, is overly complex for consumers, and does not take environmental aspects into account. Thus, it may not be useful for making sustainable and healthy choices for consumers. In Japan, there is a need to develop food-based dietary guidelines (FBDGs), which are already available in many other countries, taking environmental factors into account (51). The recommendations for each food will make it easier for the public population to choose sustainable, healthy foods. However, only a few countries (e.g., Sweden, Germany, Qatar, and Brazil) have developed food-based dietary guidelines that promote environmentally sustainable diets and dietary patterns (4). Importantly, Japan also needs to create a food environment that allows individuals to make sustainable and healthy choices. Efforts are needed to update the dietary guidelines mentioned above, develop FBDGs, reduce red meat or processed red meat products, tax these foods, subsidize plant-based protein foods, label menus in restaurants, and reduce single servings in restaurants and food retailers (52–56). However, for the consumer, urbanization, economics, and social globalization have made red meat and other environmentally unfriendly foods more enjoyable and accessible. In addition to reducing red meat intake, it will be important to undertake food environment interventions regarding the sustainable healthy food system that naturally lead to optimal choices (10, 57), as well as food and nutrition education (shokuiku in Japanese).

Although our analysis yielded several important findings, some limitations should be noted. First, because NHNS does not cover whole grains as a food item, this study did not investigate whole grains. Whole grains are considered to reduced risk of coronary heart disease, cardiovascular disease, and total cancer, and mortality from all causes, respiratory diseases, infectious diseases, diabetes, and all non-cardiovascular, non-cancer cause (58). Whole grain is also considered to be relatively lower environmental impacts (18). It is necessary for the NHNS to consider and revise food groups, such as by including whole grains, in future dietary intake surveys to more accurately reflect the SHDs and consider the impact on planetary health. Future research should further investigate the intake of rice and other “non-whole grains” by the Japanese population to compare the values with the global reference of the PHD. Additionally, recommendations and possible alternatives for shifting consumption of red meat and reducing its intake need to be further investigated. Finally, because the PHD does not indicate the specific type of red meat, no analysis could be performed to address this issue. Further analysis will be necessary to establish more specific PHD references on the basis of the environmental footprint of different types of red meat.

## Conclusion

According to the global reference of PHD, the current Japanese diet contains an excessive intake of red meat. However, the Japanese diet does not significantly exceed the recommended protein intake for the Japanese population, suggesting that shifting red meat to white meat, or even plant-based protein under the concept of the PHD is an environmentally friendly and healthy choice for both younger and older age groups in an aging Japanese society. Policy makers need to develop sustainable and healthy FBDGs in addition to providing food and nutrition education and developing a food environment that encourages sustainable and healthy choices.

## Data availability statement

The original contributions presented in the study are included in the article/Supplementary material, further inquiries can be directed to the corresponding author.

## Ethics statement

Ethical review and approval were not required for the study on human participants in accordance with the local legislation and institutional requirements.

## Author contributions

All of the authors made substantial intellectual contributions to the study, reviewed and approved the final version of the manuscript. MN and NN conceptualized and designed the study. MY and YI conducted the nutrition evaluation using NHNS data. MN drafted and finalized the manuscript. MY and NN critically commented on and revised the manuscript.

## Funding

This work was supported by JSPS KAKENHI (22 K02108).

## Conflict of interest

The authors declare that the research was conducted in the absence of any commercial or financial relationships that could be construed as a potential conflict of interest.

## Publisher’s note

All claims expressed in this article are solely those of the authors and do not necessarily represent those of their affiliated organizations, or those of the publisher, the editors and the reviewers. Any product that may be evaluated in this article, or claim that may be made by its manufacturer, is not guaranteed or endorsed by the publisher.
