# Supplementary material for: Current dietary intake of the Japanese population in reference to the planetary health diet-preliminary assessment
Source: Front Nutr. 2023 Apr 3;10:1116105. doi: 10.3389/fnut.2023.1116105 (PMC10106588; doi:10.3389/fnut.2023.1116105)
Supplement: Supplementary file 1 [file Table_1.pdf]

Table S1. The Japan National Health and Nutrition Survey dietary intake (from 2000 to 2015), reference of Planetary Health Diet and diet gap

Food group	2000 [d]			2005			2010			2015		
	PHD (g/day) [a]	NHNS (g/day) [b]	DG (%)[c]	PHD (g/day) [a]	NHNS (g/day) [b]	DG (%)[c]	PHD (g/day) [a]	NHNS (g/day) [b]	DG (%)[c]	PHD (g/day) [a]	NHNS (g/day) [b]	DG (%)[c]
Whole grains	N.A.	N.A.	N.A.	N.A.	N.A.	N.A.	N.A.	N.A.	N.A.	N.A.	N.A.	N.A.
Tubers or starchy vegetables	40 (0, 79)	64 (76)	162	38 (0, 76)	58 (76)	152	37 (0, 74)	54 (68)	144	38 (0, 76)	51 (64)	134
Vegetables	237 (158, 474)	316 [e]	133	229 (153, 459)	325 [e]	142	223 (149, 446)	311 [e]	140	228 (152, 456)	321 [e]	141
Fruits	158 (79, 237)	122 (139)	77	153 (76, 229)	127 (157)	83	149 (74, 223)	104 (129)	70	152 (76, 228)	112 (137)	74
Dairy foods	198 (0, 395)	98 (129)	50	191 (0, 382)	98 (134)	51	186 (0, 372)	92 (123)	49	190 (0, 380)	110 (137)	58
Protein sources												
Meat	N.A.	76 (70)	N.A.	N.A.	78 (72)	N.A.	N.A.	80 (71)	N.A.	N.A.	89 (77)	N.A.
Beef, lamb, and pork	11 (0, 22)	N.A.	N.A.	11 (0, 21)	N.A.	N.A.	10 (0, 21)	N.A.	N.A.	11 (0, 21)	62 (63)	587
Chicken and other poultry	23 (0, 46)	N.A.	N.A.	22 (0, 44)	N.A.	N.A.	22 (0, 43)	N.A.	N.A.	22 (0, 44)	25 (50)	114
Eggs	10 (0, 20)	39 (36)	383	9.9 (0, 19)	34 (35)	341	9.7 (0, 19)	35 (34)	362	9.9 (0, 19)	35 (36)	359
Fish	22 (0, 79)	101 (87)	455	21 (0, 76)	91 (80)	423	21 (0, 74)	78 (73)	376	21 (0, 76)	74 (71)	349
Legumes	59 (0, 79)	74 (76)	125	57 (0, 76)	62 (75)	108	56 (0, 74)	59 (74)	106	57 (0, 76)	64 (78)	112
Nuts	40 (0, 59)	2.0 (7.9)	5.1	38 (0, 57)	2.0 (6.7)	5.2	37 (0, 56)	2.2 (7.7)	5.9	38 (0, 57)	2.5 (7.7)	6.6
Added fats												
Oils and fats	N.A.	16 (14)	N.A.	N.A.	10 (9.5)	N.A.	N.A.	10 (9.1)	N.A.	N.A.	11 (9.5)	N.A.
Unsaturated oils	32 (16, 63)	N.A.	N.A.	31 (15, 61)	N.A.	N.A.	30 (15, 59)	N.A.	N.A.	30 (15, 61)	9.4 [f]	31
Saturated oils	9.3 (0, 9.3)	N.A.	N.A.	9.0 (0, 9.0)	N.A.	N.A.	8.8 (0, 8.8)	N.A.	N.A.	9.0 (0, 9.0)	1.2 [g]	13
Added sugars	24 (0, 24)	10 (11)	40	24 (0, 24)	7.3 (10)	31	23 (0, 23)	7.0 (9.2)	30	24 (0, 24)	6.9 (9.0)	29

PHD: Planetary Health Diet; NHNS: National Health and Nutrition Survey; DG: diet gap; N.A.: not available.

[a] Average dietary intake and its possible range of each food group against the 2,500 (kcal/day) in Planetary Health Diet was converted by the NHNS total energy intake (1,975 kcal/day in 2000, 1,912 kcal/day in 2005, 1,859 kcal/day in 2010, and 1,898 kcal/day in 2015).

[b] Average (standard deviation) dietary intake was referenced by the NHNS across four surveys (n = 9,676 in 2000, n = 7,262 in 2005, n = 7,229 in 2010, and n = 6,172 in 2015).

[c] Diet gap (%) = (NHNS dietary intake (g/day)/ PHD macronutrient intake (g/day)) * 100

[d] Amount of intake (g/day) in 2000 was calculated only with dried or uncooked food.

[e] Average (standard deviation) of vegetables was 294 (160) in vegetables, 16 (27) in mushrooms, 6.1 (13) in seaweeds in 2000; 293 (176) in vegetables, 17 (29) in mushrooms, 15 (33) in seaweeds in 2005; 282 (177) in vegetables, 18 (29) in mushrooms, 12 (22) in seaweeds in 2010; and 294 (177) in vegetables, 17 (27) in mushrooms, 11 (20) in seaweeds in 2015.

[f] Average (standard deviation) of unsaturated oils was 1.1 (3.5) in margarine and 8.3 (8.2) in vegetable oils.

[g] Average (standard deviation) of saturated oils was 1.0 (3.1) in butter and 0.2 (1.2) in animal fats.
